# Autophagy and Lc3-Associated Phagocytosis in Zebrafish Models of Bacterial Infections

**DOI:** 10.3390/cells9112372

**Published:** 2020-10-29

**Authors:** Salomé Muñoz-Sánchez, Michiel van der Vaart, Annemarie H. Meijer

**Affiliations:** Institute of Biology Leiden, Leiden University, Einsteinweg 55, 2333 CC Leiden, The Netherlands; s.munoz.sanchez@biology.leidenuniv.nl (S.M.-S.); m.van.der.vaart@biology.leidenuniv.nl (M.v.d.V.)

**Keywords:** innate immunity, autophagy, LAP, Dram1, p62, Optn, Cyba, Rubcn, tuberculosis zebrafish

## Abstract

Modeling human infectious diseases using the early life stages of zebrafish provides unprecedented opportunities for visualizing and studying the interaction between pathogens and phagocytic cells of the innate immune system. Intracellular pathogens use phagocytes or other host cells, like gut epithelial cells, as a replication niche. The intracellular growth of these pathogens can be counteracted by host defense mechanisms that rely on the autophagy machinery. In recent years, zebrafish embryo infection models have provided in vivo evidence for the significance of the autophagic defenses and these models are now being used to explore autophagy as a therapeutic target. In line with studies in mammalian models, research in zebrafish has shown that selective autophagy mediated by ubiquitin receptors, such as p62, is important for host resistance against several bacterial pathogens, including *Shigella flexneri*, *Mycobacterium marinum*, and *Staphylococcus aureus*. Furthermore, an autophagy related process, Lc3-associated phagocytosis (LAP), proved host beneficial in the case of *Salmonella* Typhimurium infection but host detrimental in the case of *S. aureus* infection, where LAP delivers the pathogen to a replication niche. These studies provide valuable information for developing novel therapeutic strategies aimed at directing the autophagy machinery towards bacterial degradation.

## 1. Introduction

In the last two decades, it has emerged that autophagy plays a myriad of roles in the immune system, in addition to its long known role as a degradation and recycling process essential for cellular homeostasis [1,2,3]. Autophagy represents an efficient effector mechanism of the innate immune system that directs intracellular microbes or damaged phagosomes towards degradation. In this process, microbes are trapped by cytoplasmic fragments of membranes (phagophores) that form double membrane vesicles (autophagosomes), which subsequently fuse with lysosomes to degrade the contents [4,5]. This antimicrobial autophagy response is induced upon the detection of microbial invaders by pattern recognition receptors (PRRs), such as the Toll-like and Nod-like receptors [6]. Vice versa, autophagy promotes the delivery of pathogen-associated molecular patterns (PAMPs) from the cytoplasm to PRRs in endosomes and thereby augments the innate immune response [7]. Furthermore, autophagy can function as a secretory process that contributes to the secretion of proinflammatory cytokines (such as IL-1β), but may also lead to extrusion and spreading of pathogens [8]. Autophagy regulates inflammation by suppressing inflammasome activation, type I interferon signaling, and nuclear factor κB (NFκB) signaling [2]. Finally, autophagy influences the development of immune cells [2,9,10] and it forms a link between innate and adaptive immunity by processing peptides for antigen presentation [11]. The discovery of these autophagy-dependent mechanisms has deeply changed our understanding of the immune mechanisms involved in antimicrobial host defense.

**Figure 1:**
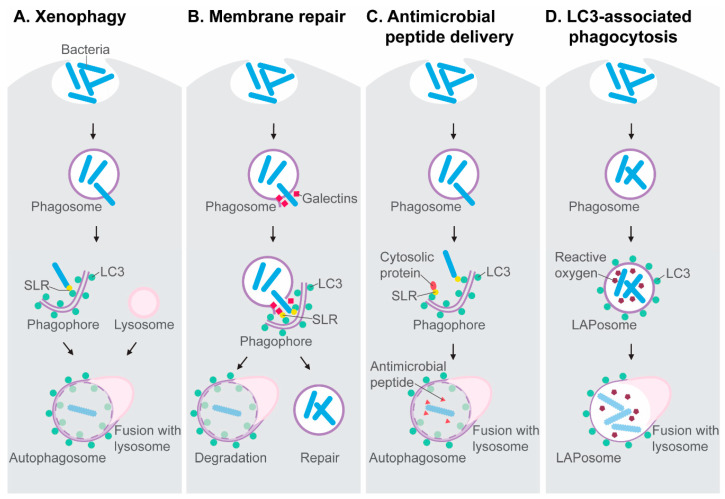
Antimicrobial mechanisms dependent on the autophagy machinery. (**A**) Xenophagy is a subtype of selective autophagy mediated by Sequestosome1-like receptors (SLRs) that target ubiquitinated microbes in the cytosol and interact with LC3 on nascent autophagosomes. (**B**) SLRs also detect galectins on damaged phagosomes, which may induce the autophagic degradation of these vesicles or initiate an autophagy-mediated membrane repair process, preventing microbes from entering the cytosol. (**C**) The autophagolysosomal pathway processes ubiquitinated proteins into antimicrobial peptides. (**D**) The formation of LAPosomes, where LC3 associates with phagosomes, is an autophagy-related process linked with reactive oxygen production.

The molecular mechanisms involved in autophagy activation and autophagosome formation have been extensively covered in other reviews [12,13,14]. Here, we briefly introduce the molecular players relevant to the antimicrobial autophagy research discussed in this review. The autophagy-mediated capture and destruction of microbial invaders is a receptor-mediated process, known as xenophagy (Figure 1A). Xenophagy is a type of selective autophagy that targets intracellular pathogens when they gain access to the cytosol of their host cell, where they become a substrate for ubiquitin E3 ligases. These enzymes tag microbial invaders with ubiquitin, which functions as an eat-me signal for Sequestosome1-like receptors (SLRs) [13,15,16]. SLRs, named after the founding member Sqstm1/p62, interact with the lipidated form of the LC3 protein, which is conjugated to autophagy membranes. As such, the microbial cargo is directed to the autophagosomal-lysosomal pathway. SLRs, notably NDP52, also detect membrane damage inflicted by bacteria when they attempt to escape phagosomes. The rupture of phagosomes exposes sugars on the phagosome membrane, which can be recognized by galectins [17]. The detection of membrane damage either directs the damaged vesicle towards degradation or it induces autophagy-mediated membrane repair (Figure 1B), delaying the bacterial escape [18,19]. Notably, SLRs, like p62, also collect ubiquitinated host proteins from the cytosol, which can be processed into antimicrobial peptides after fusion with lysosomes. This antimicrobial peptide delivery mechanism has been proposed to endow autophagosomes with a bactericidal capacity that is stronger than that of the anti-microbial environment inside phagosomes [20] (Figure 1C). 

An autophagy-related process, named LC3-associated phagocytosis (LAP), also contributes to host defense. During LAP, LC3 is recruited to the membrane of the phagosome, resulting in an LC3-decorated vesicle that is called a LAPosome [21] (Figure 1D). The discovery of LAP has emphasized that LC3 cannot be regarded as an exclusive marker of double membrane autophagosomes but can also mark single membrane phagosomes. LAP requires most of the proteins of the core autophagy machinery, such as ATG5 and ATG7, but LAP occurs independently from the components of the autophagy preinitiation complex, such as ATG13. The signaling protein Rubicon has been identified as a molecular switch that inhibits autophagy, while promoting LAP [22]. In the process of LAP, Rubicon has been found to stabilize the NADPH oxidase on phagosomal membranes, which facilitates LC3 recruitment [23]. Thus, LAP is tightly coupled to the production of reactive oxygen species by NADPH oxidase. Like the delivery of antimicrobial peptides in the autophagolysosomal pathway [20], LAP may lead to a microbicidal capacity exceeding that of the conventional phagolysosomal environment. 

Many studies demonstrate that autophagy and LAP are effective host defense mechanisms against a variety of pathogens [4,24]. However, pathogens have also evolved virulence mechanisms to either escape detection by the autophagy machinery or to subvert this system and use autophagic compartments or LAPosomes as a replication niche [25,26,27,28]. The cellular mechanisms underlying autophagy and LAP as well as the evasion strategies of pathogens have been discussed in several recent reviews cited above. Here we focus on the use of zebrafish as a convenient vertebrate model to visualize interactions of bacterial pathogens with the autophagy machinery in vivo, and increase understanding of host-protective and host-detrimental responses of the autophagy machinery.

## 2. Zebrafish Toolbox for In Vivo Study of Autophagic Defenses 

The zebrafish (*Danio rerio*) is increasingly used as model species for research into vertebrate development and disease [29]. The early life stages (embryos and larvae) of the zebrafish are easily accessible due to their external fertilization. They provide several experimental advantages, such as efficient genetic manipulation, high-throughput drug screening, and high-resolution intravital imaging with the availability of many fluorescent lines to monitor signaling pathways and track proteins, cells, organs, and tissues. Humans and zebrafish have similar innate and adaptive immune systems [30,31]. In both organisms, innate immunity is the first to develop. It is already functional in one-day-old zebrafish embryos, making it possible to study the first encounter of pathogens with the innate immune system in vivo [32]. Macrophages and neutrophils are the primary innate immune cell types during the embryonic and larval stages, which makes the zebrafish a convenient model to study encounters of pathogens with these phagocytes. A wide variety of zebrafish models for human infectious diseases have been developed and their use has revealed fundamental new insights into host–pathogen interaction mechanisms, as reviewed elsewhere [33,34,35].

Zebrafish models have been used to study the role of autophagy during development and during diseases such as cancer, neurodegeneration and infection [36,37,38]. It has been shown that embryos (0–3 days) and early larvae (3–5 days) have low basal levels of autophagy and that the transition from yolk feeding to independent feeding (at 5 days) induces a strong increase in autophagy activity [39]. Genetic knockdown of autophagy genes has been accomplished using antisense morpholino oligonucleotides or Crispr/Cas9 technology and is associated with developmental aberrations, for example defects in cardiac development [36,37,38]. However, by titrating the dose of these knockdown reagents, partial knockdown effects can be achieved, which are often sufficient to determine the role in a specific developmental or disease process. In addition, it has been shown that autophagy can be modulated in zebrafish by simple bath exposure of embryos or larvae to autophagy inducers (e.g., rapamycin, torin, Ar12, carbamazepine) or inhibitors (e.g., 3-methyladenine, chloroquine) that are widely used in mammalian research models [36,37,38,40]. 

**Figure 2:**
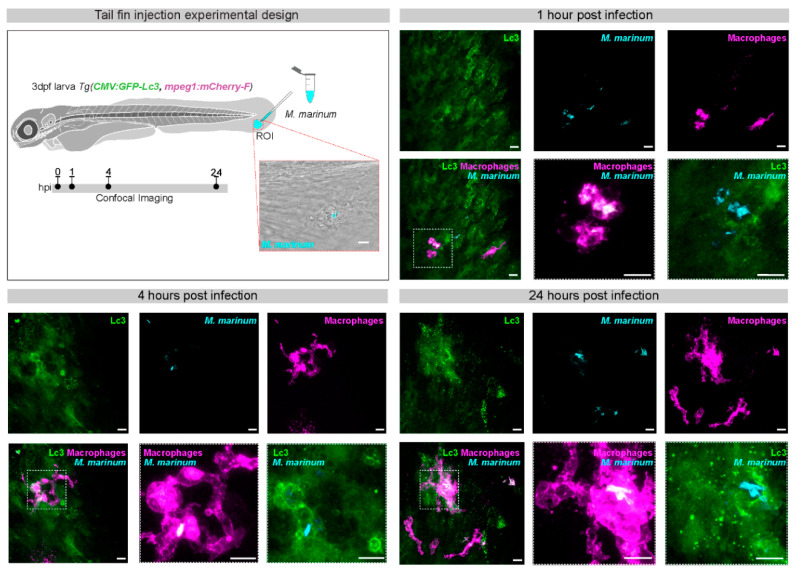
GFP-Lc3 response to *M. marinum* infection in the tail fin of zebrafish larvae. In the experimental setup of tail fin injections, mCrimson-labeled *M. marinum* bacteria (200 colony forming units) are microinjected into the tail fin area of zebrafish larvae at 3 days post fertilization (dpf), derived from a double transgenic line that contains a ubiquitously expressed *CMV:GFP-Lc3* construct and an *mpeg1:mCherry-F* construct that labels macrophages. The injection site and region of imaging (ROI) is indicated in the schematic drawing of the larva and a representative bright field and fluorescence overlay image shows the infected area in the tail fin. Confocal laser-scanning microscopy images were acquired 1, 4, and 24 h post infection (hpi). Fluorescent excitation in the range of GFP, far-red and red were used to visualize GFP-Lc3, *M. marinum,* and macrophages, respectively. At 1 hpi, macrophages are already recruited at the site of infection and phagocytosis of bacteria can be observed. At 4 hpi, GFP-Lc3 signal is observed inside infected macrophages and often appears in ring-shaped patterns. At 24 hpi, the bacterial cluster sizes and numbers are increased, both intra- and extracellularly, which is associated with strong punctate GFP-Lc3 signal in the infected tissue. The boxed areas in the representative images of the different time points are shown in detail on the right. Scale bars: 10 μm.

The possibility for imaging of autophagy at whole organism level is arguably the most important addition of the zebrafish model to the autophagy field (Figure 2). A GFP-Lc3 transgenic zebrafish line, developed in the Klionsky laboratory, has been widely applied for this purpose, often in combination with LysoTracker dyes to detect acidification of bacteria-containing compartments [41,42,43,44,45]. This transgenic line ubiquitously expresses GFP-Lc3 under the cytomegalovirus (CMV) promoter. It shows a diffuse basal fluorescent signal when autophagy levels are low, whereas intensely fluorescent GFP-Lc3 puncta can be observed when autophagy is activated. Treatment with a panel of autophagy inducers and inhibitors has been used to validate the GFP-Lc3 line and a less frequently used GFP-Gabarap line, reported in the same study [41]. During the process of maturation and lysosome fusion, autophagosomes progressively acidify and gradually lose the GFP-Lc3 signal. To follow this process of autophagic flux, tandem RFP-GFP-Lc3 reporters can be used, where the increasing ratio of RFP (acid-insensitive) to GFP (acid-sensitive) intensity provides a measure of flux. Recently, a zebrafish line was developed that expresses the RFP-GFP-Lc3 reporter specifically in neutrophils [27,46]. Furthermore, a zebrafish line expressing a GFP-LC3-RFP-LC3ΔG flux reporter has been developed, in which RFP-LC3ΔG is cleaved off from GFP-LC3 by the endogenous ATG4 protease and serves as an internal control because it localizes in the cytosol [40]. This reporter behaved similar in the zebrafish transgenic fish line as in mammalian cells, and it proved useful for the screening of autophagy-modulating compounds [40]. Finally, a neutrophil-specific p62 reporter line has been developed to visualize the targeting of pathogens by xenophagy [46]. Developing also macrophage-specific autophagy reporter lines could further facilitate the imaging of host–pathogen interactions in the zebrafish model, because this will avoid interfering fluorescence from non-immune cells.

Recent reviews have provided comprehensive overviews of the methods and reagents used for autophagy research in zebrafish [36,37,38]. Below we discuss the recent use of the zebrafish model to study autophagy and LAP responses to four bacterial pathogens, *Shigella flexneri*, *Mycobacterium marinum*, *Salmonella* Typhimurium, and *Staphylococcus aureus*, of which the latter three have been studied in our laboratory. Together, these studies in zebrafish infection models have strengthened the evidence for the innate host defense function of autophagic processes. However, the case of *S. aureus* illustrates how the autophagy machinery may also work to the benefit of the pathogen.

## 3. *Shigella flexneri*

*Shigella* strains are genetically closely related to *Escherichia coli*. Among them, *S. flexneri* is the most prevalent cause of dysentery in humans [47]. *Shigella* bacteria are translocated by microfold cells through the gut epithelium into the submucosal tissue, where their ingestion by macrophages elicits an inflammatory response. Bacteria that are released from dying cells force their entry into the basolateral side of gut epithelial cells by stimulating macropinocytotic uptake. *Shigella* then lyses the vacuole to gain access to the cytosol, where actin-based motility is acquired that enables cell-to-cell spreading [47]. 

The cytosolic residence of *Shigella* requires that this pathogen defends itself against xenophagy. Indeed, the virulence factor IcsB of *Shigella* is known to block a binding site for the autophagy protein ATG5 on another *Shigella* virulence factor, IcsA, which is required for adhesion to epithelial cells and the recruitment of actin tails. Likewise, IcsB is able to inhibit LAP [48]. Despite the inhibitory effect of IcsB, the autophagic defenses are still able to limit *Shigella* intracellular growth. Septins, a component of the cytoskeleton, work in concert with the autophagy machinery in defense against *Shigella* [49]. Trapping the bacteria within septin cages targets them for autophagic destruction [50]. Due to the fact that this process is counteracted by IcsB, the effectiveness of the defense response is a delicate balance between the autophagic activity of the host and the inhibitory virulence mechanisms of the pathogen.

A zebrafish embryo model for infection with *S. flexneri* was established to enable microscopy imaging studies superior to those achievable in mammalian models [51,52]. The zebrafish model was successfully used to demonstrate the significance of septins and antibacterial autophagy in a living organism [51,53]. Embryos were infected intravenously to study the interaction of *Shigella* with macrophages and neutrophils. These studies revealed a scavenger role for neutrophils in eliminating dead infected macrophages. Several critical events in *Shigella* pathogenesis could be captured, including escape into the cytosol, formation of septin cages, and association with the autophagy marker GFP-Lc3. The autophagy response was confirmed by ultrastructural demonstration of double membrane vesicles surrounding *Shigella* [51]. 

To provide functional evidence for the antibacterial function of autophagy in the zebrafish-*Shigella* model, a knockdown study of the ubiquitin receptor p62 was performed [51]. Indeed, growth of *Shigella* and susceptibility of the zebrafish host to the infection were increased when the selective autophagy pathway was inhibited in the absence of p62. However, autophagy could not be elevated to a host-protective level, as rapamycin, an autophagy inducer widely used to limit bacterial growth in cell-based studies, had a harmful effect on zebrafish embryos. The same observation was made in research into autophagy of *M. marinum* in zebrafish embryos [43]. These studies indicate that therapeutic modulation of antibacterial autophagy will require more specific chemical activators than the common mTOR inhibitor, rapamycin, which has strong immunosuppressive activity and induces a wide range of side effects.

## 4. *Mycobacterium marinum*

Infection of zebrafish with *M. marinum* is used as a model for human tuberculosis [33,54,55]. *M. marinum* is a natural pathogen of fish and other cold-blooded animals. It causes disease symptoms in zebrafish resembling primary characteristics of *M. tuberculosis* infection in humans. Most notably, these symptoms include the formation of immune cell aggregates called granulomas, which contain the bacterial infection either in a progressively expanding or in a dormant state. The main strength of zebrafish embryo and larval models for mycobacterial infection is the optical access to the earliest stages of granuloma formation. Imaging granuloma development in zebrafish embryos has revealed that these structures are initiated by infected macrophages and that granuloma progression is driven by recruitment of new macrophages that phagocytose the remains of dead infected macrophages [56]. Furthermore, macrophages egressing from initial granulomas seed the formation of secondary granulomas [56]. Thus, macrophages contribute to the spreading of mycobacterial infection, but they also limit intracellular mycobacterial growth through pathogen recognition and downstream activation of defense mechanisms, including autophagy [43].

Many studies have shown that autophagy induction in human or murine macrophages infected with *M. tuberculosis* promotes bacterial killing [3,57,58]. However, pathogenic mycobacteria are capable of inhibiting the entire range of innate host defenses to some extent, including autophagic host defense [26,57,59]. For example, *M. tuberculosis* inhibits autophagy by inducing the expression of host microRNAs and anti-inflammatory cytokines [60,61,62,63]. In addition, a mannosylated phosphoribosyl transferase shared between *M. tuberculosis* and *M. marinum* promotes intracellular survival in macrophages by inhibiting autophagy and reactive oxygen production. Mutation of this virulence factor in *M. marinum* leads to attenuated infection in adult zebrafish [64]. In addition, the autophagy response is highly dependent on lipid virulence factors and the presence of the conserved RD1 virulence locus, which encodes the ESAT6 secretion system-1 (ESX-1) [65]. Infection with ESX-1-deficient bacteria is strongly attenuated in the zebrafish host, which is explained by the requirement of ESX-1 for escape of *Mycobacteria* into the cytosol and consequent macrophage responses and granuloma formation [66,67]. In agreement, zebrafish infections show that wild type *M. marinum* bacteria are decorated by ubiquitin and GFP-Lc3, indicative of a selective autophagy response, whereas ESX-1-deficient bacteria hardly elicit GFP-Lc3 recruitment [43,44].

*M. marinum* infection of zebrafish embryos is usually achieved by intravenous injection of bacteria [68]. The macrophages (or monocytes) in circulation phagocytose the injected dose and subsequently invade tissues. The infected cells concentrate predominantly in the ventral part of the tail, at the location of the caudal hematopoietic tissue. This is a convenient location for confocal imaging of autophagy responses in infected macrophages, used in several studies in our laboratory [42,43,44,69,70]. As an alternative, the tail fin injection model was developed to enable higher resolution imaging and facilitate correlative light and electron microscopy [71]. Injection of *M. marinum* between the epithelial layers of the very thin fin tissue induces rapid attraction of macrophages and neutrophils and the formation of a single granuloma over several days. Performing such tail fin injections in the GFP-Lc3 line revealed two types of vesicles. The most abundant type of vesicles was small in size (approximately 1 μm). These small vesicles were closely associated with bacteria, but did not contain them. A larger type of vesicle did contain bacteria and most of these larger GFP-Lc3 vesicles were positive for a lysosomal marker. Correlative light and electron microscopy confirmed association of GFP-Lc3 with double membrane vesicles and demonstrated the presence of bacteria in GFP-Lc3-positive compartments with late autophagic morphology. It might be speculated that the smaller GFP-Lc3 vesicles contribute to antimicrobial peptide delivery to bacteria-containing compartments, as has been shown in mammalian cells [20]. In line with this possibility, electron microscopy analysis of intravenously infected zebrafish embryos provided an example of an autophagosome fusing with a larger bacteria-containing compartment [43]. 

The question whether autophagy truly serves a host-protective function during complex infectious diseases has been debated [3,72]. A study in mice showed that mutations in several autophagy genes did not change the outcome of *M. tuberculosis* infection [73]. These results suggest that, under conditions where a full adaptive immune response is mounted, the contribution of autophagy to host defense is relatively minor. However, another possible interpretation is that the observed impact of autophagy gene mutations was limited in this study, because *M. tuberculosis* is efficient at evading autophagy. If that is the case, therapeutic elevation of autophagy levels could be a means to overcome autophagy inhibition by mycobacterial virulence factors [26,74]. This idea is supported by several studies in zebrafish embryos, showing a major contribution of autophagy to the control of mycobacterial infection, at least in the context of innate immunity. Inhibition of xenophagy by knockdown of the ubiquitin receptors p62 or Optineurin reduced GFP-Lc3 associations with *M. marinum* and rendered zebrafish embryos more susceptible to infection [44]. Moreover, p62 and Optineurin overexpression resulted in an increase of GFP-Lc3-*M. marinum* associations and improved resistance of the zebrafish host. It has been also been reported that clearance of *M. marinum* can be enhanced by treatment with carbamazepine, a drug that induces autophagy by cellular depletion of myo-inositol [75]. Furthermore, chemical inhibition of GABAergic signaling increased the severity of *M. marinum* infection in zebrafish, similar to that of *M. tuberculosis* infection in mice [76]. Thus, besides the classical Lc3-mediated process, autophagy activation through GABA receptors and the Lc3 analog Gabarap may also contribute to the antimycobacterial response. Together, these studies in zebrafish demonstrate the importance of autophagy for innate host defense and show that genetic or chemical boosting of autophagy enhances host resistance. 

Studying mycobacterial infection in zebrafish led to the discovery of a new link between pathogen recognition and stimulation of autophagy flux [43]. Transcriptome analysis of zebrafish larvae with mycobacterial granulomas revealed upregulation of the gene encoding DNA-damage regulated autophagy modulator 1 (Dram1), whose human counterpart (DRAM1) is associated with an interferon-inducible expression signature in tuberculosis patients [77,78]. The expression of DRAM1/Dram1 in primary human macrophages and the zebrafish model in response to mycobacterial infection relied on the MyD88 adaptor protein and NFκB transcription factor, both components of Toll-like receptor and IL-1β signaling, identifying this pathway as an infection-inducible mechanism downstream of PRRs [43]. DRAM1 is a transmembrane protein localizing to multiple organelles, including autophagosomes, but is especially abundant on lysosomes [79]. It has been shown to promote autophagic flux under different cellular stress conditions, including DNA damage-induced programmed cell death [80]. While the molecular mechanism remains to be uncovered, colocalization studies in human cells and zebrafish embryos point towards a role for DRAM1/Dram1 in promoting the fusion of autophagosomes and lysosomes with *Mycobacteria*-containing compartments [43]. In agreement, *dram1* overexpression increases GFP-Lc3 targeting of *M. marinum* and improves host resistance. Furthermore, knockdown or mutation of *dram1* impairs the resistance to *M. marinum* in zebrafish embryos, along with reductions in GFP-Lc3-positive bacteria and in the level of acidification based on Lysotracker staining [42,43]. Together, these results support that Dram1 promotes (auto)phagosome maturation. 

Autophagy not only defends against intracellular bacteria, but it also controls inflammation [2]. Indeed, excessive inflammation was detected in ATG5-deficient mice, which was likely the major cause of their hypersusceptibility to *M. tuberculosis* infection [73]. Analysis of the *dram1* mutant phenotype revealed that activation of inflammatory caspases is a major cause of the increased infection burden in this case [42]. In the absence of Dram1, macrophages are unable to restrict growth of *M. marinum* and succumb prematurely to cell death. The hypersusceptibility of *dram1* mutants could be rescued by knocking down the zebrafish homologs of caspase 11 and gasdermin D, identifying pyroptosis as the cell death mechanism of infected macrophages that accelerates infection dissemination in the absence of Dram1 [42]. Pyroptotic cell death of infected macrophages will induce a strong local inflammatory response, attracting additional immune cells to the site of infection which are likely to become infected themselves. In light of these results, strategies for therapeutic modulation of autophagy should focus on achieving a dual effect: enhancing intracellular degradation, while reducing excessive inflammation.

## 5. *Salmonella* Typhimurium

Like *Shigella*, *Salmonella* is another major cause of gastrointestinal infections. While these infections often remain localized to the gut and are resolved relatively quickly, *Salmonella* infections can become systemic when the epithelial barrier is breached and infected macrophages carry the pathogen to other locations [81]. The zebrafish embryo model for infection with *S.* Typhimurium (formally named *S. enterica* serovar Typhimurium) is particularly well suited to study the role of macrophages in systemic disease [69,82]. Ablation of macrophages severely compromises the ability of zebrafish embryos to control *S.* Typhimurium growth, whereas neutrophil ablation has only a modest effect [69]. Furthermore, the response of the autophagy machinery to *S.* Typhimurium as measured by imaging GFP-Lc3 associations occurs predominantly in macrophages [69]. 

In mammalian studies, autophagic defenses in macrophages have received little attention as compared to extensive research into the molecular mechanisms of anti-*Salmonella* autophagy in epithelial cells [83]. Xenophagy is the main process that directs *Salmonella* to degradation in epithelial cells by receptors targeting ubiquitinated bacteria and molecules like diacylglycerol and galectin-bound sugars on the membranes of damaged *Salmonella*-containing vesicles [84,85,86]. In addition, occurrence of LAP has also been detected in epithelial cells as well as in mammalian neutrophils [87]. In zebrafish macrophages, LAP appears to be the main autophagy-related process contributing to host defense [69].

The role of LAP as a host-protective mechanism in zebrafish embryos is supported by genetic interrogation of factors critical for autophagy or LAP [69]. First, knockdown of Atg5, which is required for both processes, reduced GFP-Lc3 targeting of *Salmonella* and impaired zebrafish host resistance. Second, knockdown of Atg13, which affects autophagy but not LAP, had no significant impact on GFP-Lc3 associations and host resistance. Third, GFP-Lc3 associations and host resistance were reduced by knockdown of factors required for induction of LAP, being Rubicon and the Cyba component of NADPH oxidase. The production of reactive oxygen species, which is intricately linked with LAP, was demonstrated using a bacterial biosensor that showed activity when bacteria were ingested by macrophages of wild type zebrafish but not in Rubicon- or Cyba-depleted hosts. In conclusion, these results highlight LAP as a host-beneficial mechanism that restricts *Salmonella* growth inside macrophages.

*Salmonella* expresses a large array of virulence factors and some of these have been shown to counteract the autophagic defenses, also in zebrafish. The *spv* virulence locus has been shown to inhibit autophagosome formation and is a strong determinant of *Salmonella* pathogenicity in zebrafish larvae [88,89]. The autophagy-suppressing function of the *spv* locus is an effective escape response that also dampens the type I interferon response and phagocyte recruitment [90]. The relationship between several other virulence factors and LAP has also been studied in zebrafish [70]. The *purA* locus mediates metabolic adaptation to the host environment and Δ*purA* mutant bacteria are rapidly cleared by the zebrafish host. GFP-Lc3 recruitment towards this avirulent strain was strongly reduced. However, a similar observation was made with a hypervirulent Δ*flhD* strain. This strain is deficient in development of flagella, which suggests that recognition of flagellin by zebrafish PRRs promotes the GFP-Lc3 response. These and other mutants, with the notable exception of Δ*ssrB* (a *Salmonella* Pathogenicity Island 2 mutant), all became more virulent in Rubicon-deficient zebrafish, strengthening the evidence for the host-protective function of LAP.

Collectively, the results of *Salmonella* infection studies in zebrafish support the critical role of the autophagy machinery for defense against this pathogen and indicate that this model will be useful for screening therapeutic modulators of autophagy and LAP. Showing the potential of such an approach, *trans*-resveratrol treatment increased GFP-Lc3 levels in zebrafish larvae and restricted *S.* Typhimurium growth during gastrointestinal infection [91].

## 6. *Staphylococcus aureus*

*S. aureus* is a commensal bacterial species that is usually harmless but can also evade the host defenses and cause a wide range of pathologies, from skin infections to fatal systemic disease [92]. *S. aureus* is classically categorized as an extracellular pathogen, but it has become increasingly apparent that intracellular stages in host phagocytes play a critical role in the pathology of staphylococcal infections [93,94,95]. In both mice and zebrafish models, it has been observed that *S. aureus* infections develop in a clonal manner, which means that they are often derived from a single bacterium or a small fraction of the initial inoculum [93,94]. The bacteria that are at the root of such clonal expansion appear to be those that have survived an intraphagocyte stage. Selective ablation of either macrophages or neutrophils in zebrafish embryos showed that neutrophils are the most likely candidates for a pathogen reservoir from which bacteria can eventually be released to cause disseminated infection [93].

The interaction between *S. aureus* and autophagy is complex. *S. aureus* virulence factors regulated by accessory gene regulator (*agr*) and the α-hemolysin toxin trigger autophagy activation in mammalian cells [96,97]. In different mouse fibroblast cell lines, *atg5* knockout resulted either in a decrease or an increase of intracellular *staphylococci* [96,98]. Adding to this controversy, results in mouse fibroblasts indicate that LC3-positive vesicles function as a niche for bacterial replication but host-protective effects of xenophagy have also been found. However, *S. aureus* can evade the defense function of xenophagy by activating a host MAP kinase [98]. Bacterial strains or host cell types may be responsible for the observed differences in *S. aureus*–autophagy interactions. Nevertheless, the emerging picture is that xenophagy is partially effective in restricting staphylococcal growth, but the pathogen can inhibit this response or use autophagic mechanisms to its advantage to create an intracellular niche.

Studying *S. aureus* infection in the in vivo context of the zebrafish embryo provided new evidence for both host-beneficial and host-detrimental effects of the pathogen’s interaction with the autophagy machinery and revealed differences in the responses between phagocyte cell types. GFP-Lc3 was found to associate with *S. aureus* in both macrophages and neutrophils, but in macrophages this response decreased over time, while it was persistent in neutrophils [27]. Notably, neutrophils develop spacious GFP-Lc3-positive and non-acidified vacuoles containing *S. aureus* [27]. The location of *S. aureus* in neutrophils gradually shifts from this vesicular pattern to a cytoplasmic pattern, making the bacteria a potential target for xenophagy [46]. Indeed, a fluorescent p62 reporter was found to associate with *S. aureus* and knockdown or mutation of *p62* rendered zebrafish embryos more susceptible to infection [27,46]. In contrast to this host beneficial role of xenophagy, the autophagy-related LAP response appears to be responsible for the formation of the spacious GFP-Lc3 vesicles in zebrafish neutrophils. Preventing the formation of these *S. aureus*-containing LAPosomes by chemical (diphenyleneiodonium treatment) or genetic (*cyba* knockdown) inhibition of NADPH oxidase resulted in increased host resistance to the infection, indicating that *S. aureus* employs LAPosomes as an intracellular niche. In conclusion, the model emerging from these results is that LAP and xenophagy play antagonistic roles in *S. aureus*-infected neutrophils, where LAP drives bacterial pathogenesis and xenophagy benefits the host by targeting bacteria released from LAPosomes.

## 7. Concluding Remarks

**Figure 3:**
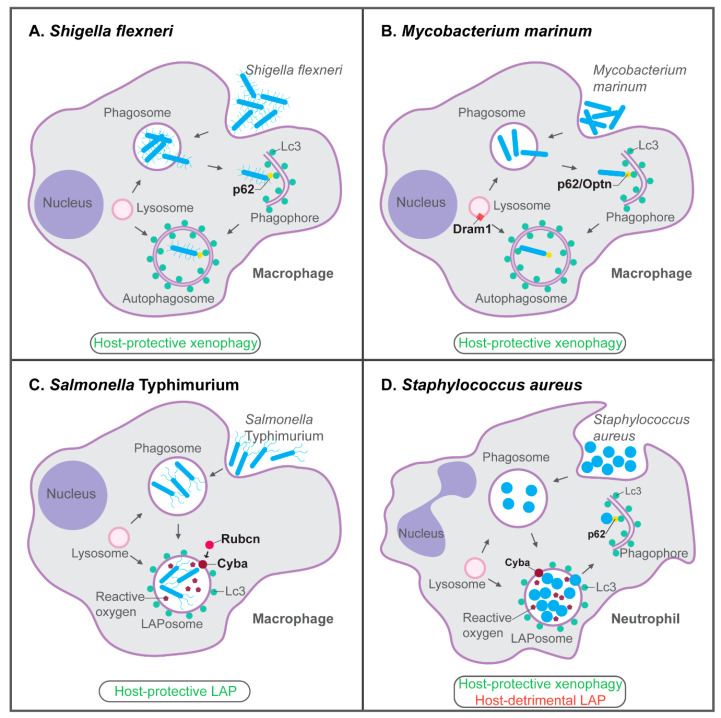
Host-protective and host-detrimental interactions of bacterial pathogens with the autophagy machinery in zebrafish infection models. (**A**) p62-dependent xenophagy restricts growth of *S. flexneri* bacteria in macrophages of the zebrafish host. (**B**) In the zebrafish *M. marinum* model, p62, Optn, and Dram1 are required for host resistance. p62 and Optn both mediate xenophagy in infected macrophages, while Dram1, an integral membrane protein of intracellular vesicles including lysosomes, promotes vesicle fusion events. (**C**) LAP, which requires the signaling molecule Rubicon (Rubcn) and the Cyba component of NADPH oxidase, is the predominant defense response of zebrafish macrophages to *S.* Typhimurium infection. (**D**) In zebrafish neutrophils, Cyba-mediated LAP provides a replication niche for *S. aureus*, while p62-mediated xenophagy counteracts the growth of bacteria that may be released from phagosomes or LAPosomes. Functions of the proteins indicated in bold have been demonstrated by knockdown, mutation, and/or overexpression analyses. See the text for references.

Zebrafish infection models are proving to be valuable additions to cell culture systems and mammalian models for studying how autophagic mechanisms contribute to the innate host defenses or underlie diseases pathologies. These models are especially useful to study the role of the autophagy machinery in macrophages and neutrophils in vivo, but also to study its systemic effects on inflammation. The studies with zebrafish embryos and larvae that we have discussed in this review provide in vivo support for the antimicrobial function of xenophagy in defense against bacterial pathogens, including *Shigella*, *Mycobacterium* and *Staphylococcus* species. Furthermore, these studies revealed opposite roles for LAP as a host protective mechanism in *Salmonella* infection versus an immune subversion mechanism in *Staphylococcus* infection (Figure 3). The host-protective LAP response towards *Salmonella* was detected predominantly in macrophages, while neutrophils emerged as the cell type where LAPosomes form a replication niche for pathogenic *Staphylococci*. The work on *Staphylococcus* illustrates how different autophagic mechanisms, xenophagy, and LAP, can operate simultaneously within the infected host. Ultimately, the outcome of infection is determined by the balance between host-beneficial and host-detrimental responses. 

Autophagy-modulating therapeutic strategies require careful consideration, taking into account the unique interactions that different pathogens may have with the autophagy machinery. It will be especially desirable to identify more specific drugs modulating the autophagy machinery, ideally those that are able to distinguish between xenophagy and LAP activation, or those that target the late stages of vesicle maturation and lysosome fusion, where pathogen degradation occurs. An attractive target for drug development is the process mediated by the infection-inducible autophagy modulator Dram1, which was discovered in zebrafish [43]. Dram1 and its human family members DRAM1 and DRAM2 are transmembrane proteins proposed to promote degradation of *Mycobacteria* by stimulating autophagic flux [43,60,99]. Considering that DRAM proteins are found on lysosomal membranes [79], it is conceivable that they stimulate not only the fusion of lysosomes with autophagosomes, but may also promote lysosomal fusion with phagosomes and LAPosomes. Recently established CRISPR/Cas9 zebrafish mutants of Dram1 and other autophagy proteins are useful tools to help dissect antimicrobial autophagy effects in future screens for novel therapeutics [44,46]. Finally, newly developed zebrafish transgenic autophagy reporter lines are powerful tools for real-time imaging studies, which will help to gain better understanding of how pathogens interact with the autophagy machinery, from their first encounter with host phagocytes until advanced stages of disease progression. These studies will facilitate the informed design of autophagy-modulating strategies tailored towards specific pathogens.
